# Ferredoxins: Functions, Evolution, Potential Applications, and Challenges of Subtype Classification

**DOI:** 10.3390/cimb46090574

**Published:** 2024-09-01

**Authors:** Khajamohiddin Syed

**Affiliations:** Department of Biochemistry and Microbiology, Faculty of Science, Agriculture and Engineering, University of Zululand, KwaDlangezwa, Empangeni 3886, South Africa; syedk@unizulu.ac.za; Tel.: +27-(035)-902-6857

**Keywords:** ferredoxin, evolution, Fe-S cluster proteins, lateral gene transfer, classification, catalysis

## Abstract

Ferredoxins are proteins found in all biological kingdoms and are involved in essential biological processes including photosynthesis, lipid metabolism, and biogeochemical cycles. Ferredoxins are classified into different groups based on the iron-sulfur (Fe-S) clusters that they contain. A new subtype classification and nomenclature system, based on the spacing between amino acids in the Fe-S binding motif, has been proposed in order to better understand ferredoxins’ biological diversity and evolutionary linkage across different organisms. This new classification system has revealed an unparalleled diversity between ferredoxins and has helped identify evolutionarily linked ferredoxins between species. The current review provides the latest insights into ferredoxin functions and evolution, and the new subtype classification, outlining their potential biotechnological applications and the future challenges in streamlining the process.

## 1. Introduction

Ferredoxins are a large class of iron-sulfur (Fe-S) cluster containing proteins that are found across all the domains of life [1]. Ferredoxins play crucial roles in many fundamental biological processes including photosynthesis, lipid metabolism, cellular respiration, and the biogeochemical cycles of hydrogen, nitrogen, and sulfur [2,3,4,5], Fe-S cluster synthesis, steroidogenesis, bile acid production, and vitamin metabolism [6]. The involvement of ferredoxins in oxidation-reduction processes makes them essential proteins in organisms ranging from non-photosynthetic anaerobic bacteria to photosynthetic unicellular and multicellular life forms [3,6]. It is interesting to note that, apart from their primary role in electron transfer, ferredoxins are also found to have regulatory functions such as enhancing the expression of genes involved in photooxidative stress [7], remodeling of regulatory protein complexes [8], controlling cellular posttranslational lipoylation [9], and controlling copper-dependent cell death (cuproptosis) [10]. 

Ferredoxins were discovered in 1962 in the obligate anaerobic non-photosynthetic bacterium, *Clostridium pasteurianum* [11]. Due to their unique property of containing only iron and not heme or flavin cofactors, and for being able to transfer electrons to many protein redox partners, authors named them “ferredoxins” [11]. Based on the rudimentary function of electron transfer and internal sequence symmetry, ferredoxin proteins are thought to have evolved during abiogenesis in the burgeoning Earth [2,12,13,14,15,16]. Thus, these proteins are considered living protein fossils [2,12,13,14,15,16]. A well-known hypothesis is that ferredoxins evolved through tandem gene duplications encoding smaller proteins, which may have originated from duplicating even simpler ancestral peptides [11,12,13], and a recent study provided further evidence strengthening this hypothesis [1].

## 2. Ferredoxin Functions

Ferredoxins are involved in a range of biological processes, and any disruption of electron transfer from ferredoxins eventually disrupts that biological process. Important roles of ferredoxins in various biological processes include: 

### 2.1. Pyruvate Synthesis/CO_2_ Fixation 

**Figure 1:**
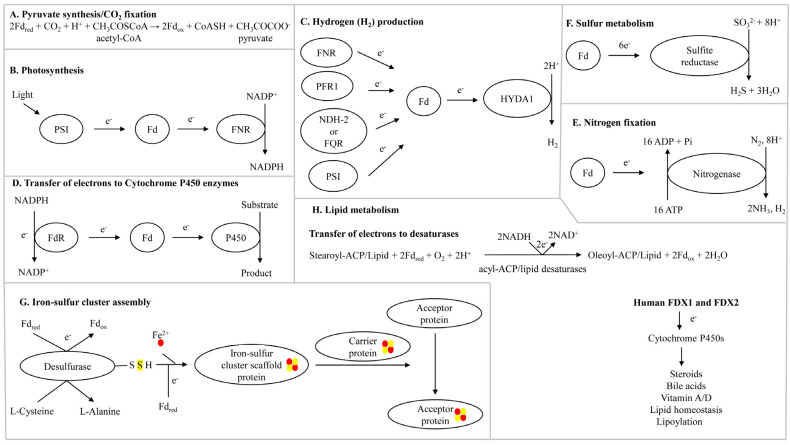
Schematic diagram showing ferredoxin’s role in various biological processes. Abbreviations: PSI, photosystem I; FNR, ferredoxin-NADP+ reductase; FQR, ferredoxin-plastoquinone reductase; NDH-2, NAD(P)H-PQ oxidoreductase; PFR1, pyruvate:ferredoxin oxidoreductase; HYDA1, [FeFe]-hydrogenase; FdR, ferredoxin reductase; Fd, ferredoxin; e^−^, electrons; ATP, adenosine triphosphate; Pi, phosphate; SO_3_^2−^, sulfite; H_2_S, hydrogen sulfide; CO_2_, carbon dioxide; H^+^, hydrogen ion; CoASH, coenzyme A; Fd_ox_ and Fe_red_, ferredoxin-oxidized and ferredoxin-reduced; FDX1 and FDX1, human mitochondrial ferredoxins 1 and 2. Iron and sulfur atoms are presented with red and yellow dots.

The acetyl-CoA pathway is considered an ancient pathway for CO_2_ fixation that evolved >3.8 billion years ago in the autotrophs, where acetyl-CoA is converted to pyruvate by ferredoxin-dependent CO_2_ fixation [17] (Figure 1A). In this reaction, ferredoxins are directly reduced by hydrogen via native iron or flavin-based hydrogenases [17]. The role of ferredoxins in transferring electrons is key in pyruvate biosynthesis and its further bioconversion.

### 2.2. Role in Photosynthesis

In photosynthetic organisms, ferredoxins accept electrons from photosystem I (PSI) and transfer them to ferredoxin-NADP+ reductase (FNR) (Figure 1B). During the electron transfer, NADPH is produced (Figure 1B). The produced NADPH is then utilized for the production of ATP. 

### 2.3. Role in the Production of Hydrogen

Hydrogen is a clean energy source, and producing this valuable gaseous molecule from organisms is an active area of research. Ferredoxins play a vital role in the production of this clean energy [18,19]. The production of hydrogen by the green alga *Chlamydomonas reinhardtii* has been well-studied, and the role of ferredoxin is well-defined [18]. Multiple pathways in *C. reinhardtii* that can transfer electrons to ferredoxins have been identified [18]. A specific ferredoxin (PetF) receives electrons from multiple donor partners under different conditions including PSI, FNR, NAD(P)H-PQ oxidoreductase (NDH-2), ferredoxin-plastoquinone reductase (FQR), or pyruvate:ferredoxin oxidoreductase (PFR1) (Figure 1C). The reduced ferredoxin subsequently transfers electrons to [FeFe]-hydrogenase HYDA1, which further catalyzes the reduction of protons into H_2_ (Figure 1C) [18].

### 2.4. Transfer of Electrons to Cytochrome P450 Monooxygenases

Cytochrome P450 monooxygenases (CYPs/P450s) perform various oxidative enzymatic reactions important in biology. P450s require electrons to perform their enzymatic activity, and ferredoxins transfer these electrons, particularly in bacterial P450 systems [20,21]. Ferredoxins not only transfer electrons to P450s but also modulate their functions; thus, the factors governing their molecular interactions are crucial for the successful outcome of the catalyzed reaction [20]. The P450 catalytic cycle starts with ferredoxin reductases (FdRs) extracting electrons from NADPH and reducing ferredoxins (Figure 1). The reduced ferredoxins then transfer the electrons to P450s in order to catalyze the molecular scission of atmospheric dioxygen (Figure 1D). Information on the transfer of electrons by ferredoxins to different mitochondrial human P450s that play a role in steroid metabolism (Figure 1D) has been reviewed elsewhere [5].

### 2.5. Role in Nitrogen Fixation

Specific microbes including cyanobacteria, rhizobia, green sulfur, and purple sulfur bacteria convert atmospheric nitrogen (N_2_) into bioavailable ammonia (NH_3_), which is essential for sustaining life [22]. Microbes use nitrogenase and ferredoxin enzymes to fix atmospheric nitrogen [23] (Figure 1E). Ferredoxins transfer electrons to nitrogenase, which converts N_2_ into NH_3_. During this reaction, 16 ATP molecules are utilized, and hydrogen is produced as a by-product [23] (Figure 1E).

### 2.6. Role in Sulfur Metabolism

Ferredoxins play a role in sulfur metabolism in archaea, bacteria, fungi, and plants. For example, they transfer electrons to sulfite reductase, which converts sulfite into hydrogen sulfide and water [24,25] (Figure 1F). This is an essential process for synthesizing sulfur-containing compounds in the cell.

### 2.7. Role in Iron-Sulfur Cluster Biosynthesis

The role of ferredoxins in transferring electrons for the biogenesis of iron-sulfur clusters have been elucidated [26,27] (Figure 1G). The mechanism of iron-sulfur cluster assembly has been studied in detail in cyanobacteria and humans [26,27]. The generally accepted pathway for iron-sulfur cluster biosynthesis can be divided into two main steps: (1) the assembly of the iron-sulfur cluster on a scaffold protein using iron and sulfur atoms and (2) the transfer of the iron-sulfur cluster to the acceptor protein [26,27] (Figure 1G). Two ferredoxins from human mitochondria (FDX1 and FDX2) have been shown to be involved in transferring electrons to iron-sulfur cluster assembly proteins, but FDX2 was found to transfer electrons at a faster rate than FDX1 [26] (Figure 1G). The reduced ferredoxins successfully transferred electrons to the iron-sulfur cluster assembly proteins, particularly cysteine desulfurases, whereby L-cysteine is converted to L-alanine with the simultaneous generation of sulfide [26] (Figure 1G). Reduced ferredoxins supported the iron-sulfur cluster assembly on the iron-sulfur cluster scaffold protein [26] (Figure 1G). 

### 2.8. Role in Lipid Metabolism

Ferredoxins play essential roles in lipid metabolism [6]. For example, ferredoxins transfer electrons to acyl-ACP and acyl-lipid desaturases, leading to the generation of unsaturated fatty acids in plants and cyanobacteria [28,29] (Figure 1H).

Human FDX1 is involved in various processes associated with lipid metabolism, such as in the biogenesis of steroids and bile acids, vitamin A/D metabolism, and lipoylation of tricarboxylic acid (TCA) cycle enzymes [5,6] (Figure 1H). A thorough analysis of FDX1’s role has been previously reviewed [5] and so these processes are not elaborated in the present review. A study in mice revealed that FDX1 is essential for mammalian embryonic development and lipid homeostasis, and FDX1 deficiency led to the alteration of several classes of sterols and lipids, including cholesterol, triacylglycerides, acylcarnitines, ceramides, phospholipids, and lysophospholipids [6]. 

## 3. Classification of Ferredoxins-Types

**Figure 2:**
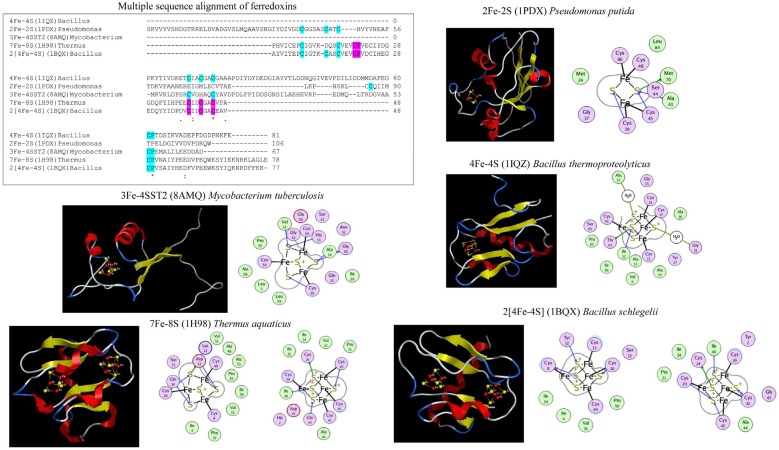
Ferredoxin interactions with different iron-sulfur (Fe-S) clusters. Each ferredoxin type is presented with its type name, Protein Databank (PDB) identification number in parenthesis, and species name. The ferredoxin crystal structures include 2Fe-2s (1PDX) from *Pseudomonas putida* [32], 3Fe-4S (8AMQ–ferredoxin only) from *Mycobacterium tuberculosis* H37Rv [33], 4Fe-4S (1IQZ) from *Bacillus thermoproteolyticus* [34], 7Fe-8S (1H98) from *Thermus aquaticus* [35], and 2[4Fe-4S] (1BQX) from *Bacillus schlegelii* [36]. In multiple sequence alignment, the characteristics of residues interacting with iron atoms, especially cysteine residues, are highlighted along with the characteristic proline residue. Amino acid residues are highlighted with two colors to distinguish interactions with two Fe-S clusters. The individual ferredoxins are shown with their crystal structure and Fe-S cluster(s) interactions. The covalent interactions between the Fe atom and cysteine residues are shown with a solid line. In the crystal structure, Fe and S are colored red and yellow.

Ferredoxins have been classified into different groups based upon the number of iron (Fe) atoms in their structure (Figure 2). These reported groups include 2Fe-2S, 3Fe-4S, 4Fe-4S, 7Fe-8S (3Fe-4S and 4Fe-4S), and 2[4Fe-4S] [30]. Each type of Fe-S cluster boasts a distinctive Fe-S sequence binding motif containing specific cysteine amino acids that coordinate with the Fe atom. The 2Fe-2S cluster type features four cysteines in its binding motif; the 3Fe-4S cluster type is three cysteines and a proline residue following the third cysteine; the 4Fe-4S cluster type consists of four cysteines and a proline following the fourth cysteine, and the 7Fe-8S ferredoxins encompass characteristics of both the 3Fe-4S and 4Fe-4S clusters (Figure 2). In the 2[4Fe-4S] ferredoxins the spacing between the cysteines binding to the Fe atom differs from that of the 4Fe-4S cluster type motif (Figure 2). The 2[4Fe-4S] proteins are bifurcated into two subfamilies: small proteins (approximately 55 amino acids) housing iso-potential Fe-S clusters and larger proteins named Alvin (Alv) ferredoxins, possessing Fe-S clusters with varying potentials [31]. Sequence analysis has uncovered an additional cysteine in 2[4Fe-4S]Alv ferredoxins, precisely three amino acids after the final cysteine of the second 4Fe-4S binding cluster [31].

## 4. Evolution of Ferredoxins 

Experiments mimicking the early conditions of Earth, such as the chemical evolution period, resulted in the formation of 4Fe-4S clusters, and thus it is now widely believed that 4Fe-4S clusters were the first to evolve among Fe-S cluster types [12,37,38]. Several lines of evidence also support these observations. 4Fe-4S clusters are more sensitive to oxygen than 2Fe-2S clusters [39,40,41]. This indicates that after the Great Oxidation Event species might have preferred 2Fe-2S clusters as they are inherently oxygen tolerant. The presence of abundant 4Fe-4S cluster-type proteins in anaerobes and 2Fe-2S cluster-type proteins in aerobes [42] strongly suggests that 4Fe-4S clusters were the first to evolve.

2[4Fe-4S] ferredoxins have a symmetrical arrangement of Fe-S binding motifs in their structure (Figure 2). Based on the symmetric arrangement of the Fe-S cluster binding motif of 2[4Fe-4S], it has been proposed that ferredoxins arose through the duplication of genes encoding even shorter and simpler ancestral peptides [16]. Many findings suggest that 2[4Fe-4S] ferredoxins have drifted from their symmetric roots via gene duplication followed by mutations ([28]). Additionally, studies report that gene duplication led to the growth and diversification of Fe-S cluster proteins [42]. 

Knowledge regarding the evolution of ferredoxins is scarce, but current data suggest that ferredoxins may have originated from a common ancestor and then undergone divergent evolution, leading to their diversity today [13,43,44,45,46]. 

## 5. Ferredoxin Subtype Classification and Nomenclature

**Figure 3:**
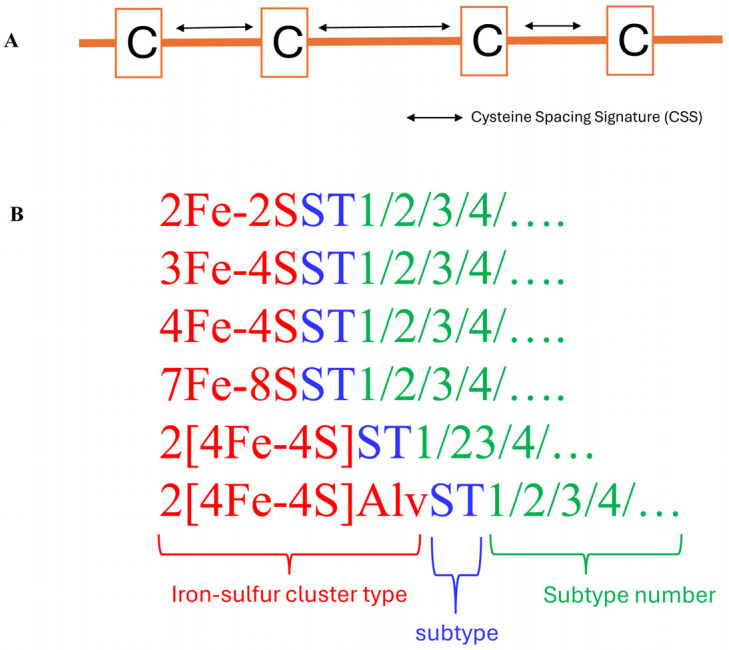
Ferredoxins subtype classification criteria (**A**) and nomenclature (**B**). (**A**). The spacing pattern between the cysteine amino acids of the ferredoxin Fe-S cluster binding motif within the same cluster type is considered a cysteine spacing signature (CSS) and assigned to a particular subtype (Table 1). Ferredoxins with the same CSS are grouped under the same subtype. (**B**). To identify ferredoxins belonging to the same subtype, a nomenclature system was developed whereby each ferredoxin was represented by its type, followed by the abbreviation ST, indicating its subtype, and a number showing it belongs to that subtype number, such that ferredoxins that belong to a subtype have the same CSS.

Ferredoxins are classified into types based on their Fe-S clusters (see Section 3, Classification of Ferredoxins types). However, this classification will only help us to observe the abundance of cluster types in a species. This classification system does not help us to understand which ferredoxin types are conserved between and among the species of prokaryotes or eukaryotes, nor the diversity of ferredoxins within the cluster types. Therefore, ferredoxin subtype classification and nomenclature was proposed [1] (Figure 3). 

Ferredoxin classification into different subtypes is based on the spacing pattern of amino acids between the conserved cysteine residues of the Fe-S cluster binding motif [1] (Figure 3). The number of amino acids between each of the cysteine residues of ferredoxin is considered a cysteine spacing signature (CSS), and is represented as the characteristic signature of a subtype [1] (Figure 3). This indicates that ferredoxins of a particular subtype have the same CSS (Table 1). To distinguish between ferredoxins belonging to the same subtype, a nomenclature system was proposed whereby a specific ferredoxin is identified by its Fe-S cluster type, followed by an ST abbreviation, indicating subtype, and a numerical number indicating its subtype [1] (Figure 3 and Table 1).

## 6. Applications of Subtype Classification

Fe-S cluster type classification does not address the diversity of ferredoxins within cluster types. Also, it does not assist in identifying ferredoxins that are evolutionarily linked across species and belonging to different domains. However, ferredoxin subtype classification revealed unparalleled diversity within the ferredoxin cluster types and helped to identify evolutionarily linked ferredoxin in prokaryotes and eukaryotes and laterally/horizontally transferred ferredoxin genes between prokaryotes and eukaryotes. 

### 6.1. Diversity Analysis/Enrichment/Evolutionary Linkage of Ferredoxins in Organisms

**Figure 4:**
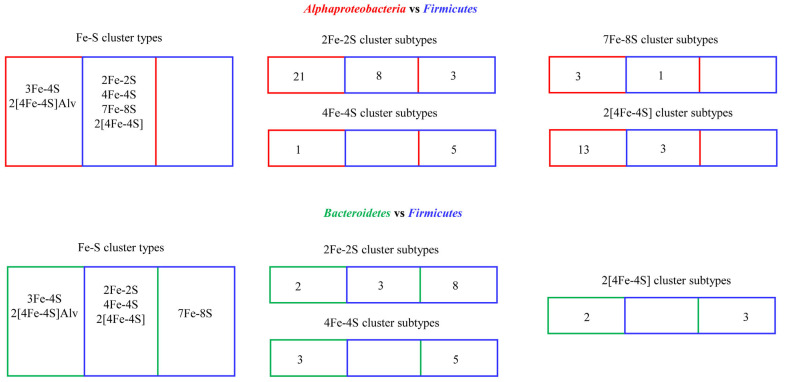
Comparative analysis of ferredoxins in *Alphaproteobacteria*, *Firmicutes*, and *Bacteroidetes*. Each number indicates subtype quantity in a particular Fe-S cluster type, as reported in the literature [1,48].

The subtype classification of ferredoxins enabled us to understand the diversity and enrichment (presence of a particular subtype of ferredoxin in organisms belonging to a specific group) of specific subtypes of ferredoxins and the evolutionary linkage of passing the same ferredoxins between species [1,48]. Analysis of ferredoxins in *Alphaproteobacteria* and *Firmicutes* revealed they share four Fe-S cluster-type ferredoxins, and two Fe-S cluster-type ferredoxins that are unique to *Alphaproteobacteria* [1] (Figure 4). Ferredoxin-subtype analysis revealed that these two groups of organisms have diverse subtypes within a particular Fe-S cluster type, and within the subtypes, a few subtypes are preferred/enriched, as these are present in many species [1]. The subtyping of ferredoxins also revealed that some ferredoxins have passed from *Alphaproteobacteria* to *Firmicutes*. Analysis of subtypes within a particular Fe-S cluster-type ferredoxin revealed that eight 2Fe-2S subtypes, three 2[4Fe-4S] subtypes, and a single 7Fe-8S subtype were shared between these two groups of organisms, indicating the common ancestral origin of these subtype of ferredoxins [1] (Figure 4). 

In general, analysis of ferredoxins and their subtypes between *Bacteroidetes* and *Firmicutes* revealed that these phyla possess highly diverse Fe-S cluster-type ferredoxins, and within the common Fe-S cluster types, ferredoxins were found to be distinct as they belong to different subtypes [48] (Figure 4). Three Fe-S cluster types (2Fe-2S, 4Fe-4S, and 2[4Fe-4S]) are commonly present between both phyla (Figure 3) [48]. Two Fe-S cluster types, 3Fe-4S and 2[4Fe-4S]Alv, are unique to *Bacteroidetes*, and one Fe-S cluster type, 7Fe-8S, is unique to *Firmicutes* [48] (Figure 4). As observed for *Alphaproteobacteria* and *Firmicutes*, *Bacteroidetes* also contain a particular Fe-S cluster type and subtype species in this phyla enrich for these ferredoxins [48]. Subtyping analysis revealed that *Bacteroidetes* and *Firmicutes* only share three subtypes of 2Fe-2S cluster-type ferredoxins, indicating their common ancestral origin [48]. However, no common 4Fe-4S and 2[4Fe-4S] subtypes were found between these two bacterial groups (Figure 4), indicating that these ferredoxins are highly diverse in bacterial phyla [48]. 

### 6.2. Lateral/Horizontal Gene Transfer (LGT/HGT) of Ferredoxins

A handful of studies reported lateral/horizontal gene transfer (LGT/HGT) of ferredoxins [8,49,50]. These studies relied on the percentage similarity between ferredoxins. The ferredoxin domain of cyanobacterial origin was acquired by photosynthetic eukaryotes (*Chlamydomonas reinhardtii*) through HGT in chloroplast DnaJ-like proteins and further passed to *Archaea* (*Nitrosopumilus maritimus* and *N. gargensis*) [8]. Eukaryotic protists such as *Giardia lamblia* and *Entamoeba histolytica* possess ferredoxins that are suggested to have been acquired from anaerobic bacteria by LGT [49]. For example, the Archean *Halobacterium salinarum* is believed to have obtained a ferredoxin by LGT from a cyanobacterial species [50]. 

**Figure 5:**
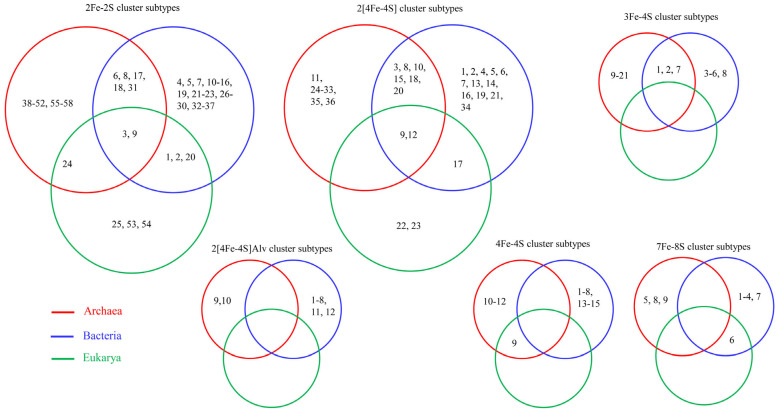
Lateral/horizontal gene transfer (LGT/HGT) of ferredoxin in domains of life. Each number indicates the subtype number in a specific Fe-S cluster-type ferredoxin. Ferredoxin subtypes identified in different domains of life, such as Archaea, Bacteria, and Eukarya, were retrieved from published articles [1,47,48].

The subtype classification of ferredoxins helps to understand and provide insights into ferredoxin LGT across domains of life [1,47,48] (Figure 5). Since ferredoxin subtyping is not based on similarity but on CSS, ferredoxins belonging to the same subtype are expected to have a common evolutionary linkage [1]. Subtypes 3 and 9 in 2Fe-2S and subtypes 9 and 12 in 2[4Fe-4S] were found to be present across the *Archaea*, *Bacteria*, and *Eukarya*, indicating ferredoxin LGT from *Archaea*/*Bacteria* to *Eukarya* (Figure 5). LGT of subtype 24 in 2Fe-2S and subtype 9 in 4Fe-4S between *Archaea* and *Eukarya* and subtypes 1, 2, and 20 in 2Fe-2S and subtype 17 in 2[4Fe-4S] between *Bacteria* and *Eukarya* was observed (Figure 5) [1,47,48]. Many subtypes, such as 6,8,17,18, and 31 in 2Fe-2S; 3,8,10,15,18, and 20 in 2[4Fe-4S]; and 1,2, and 7 in 3Fe-4S, are commonly conserved between *Archaea* and *Bacteria*, indicating the common evolutionary origin of these ferredoxins [1,47,48] (Figure 5). 

## 7. Challenges of Assigning Ferredoxins to Different Fe-S Cluster Types and Subtypes 

Many databases, such as the National Center for Biotechnology (NCBI) (https://www.ncbi.nlm.nih.gov/ (accessed on 2 August 2024)), the Universal Protein Resource (UniProt) (https://www.uniprot.org/ (accessed on 2 August 2024)), and the Kyoto Encyclopedia of Genes and Genomes (KEGG) (https://www.genome.jp/kegg/ (accessed on 2 August 2024)), list known ferredoxins in species and ferredoxins can be identified by InterPro or Pfam ID and or by manually searching the name “ferredoxin”. However many ferredoxins are still not annotated, even in well-described genome databases [1,47,48]. Thus, manual searching and further grouping of ferredoxins still remains effective. Identification of Fe-S binding-motif characteristics remains a biological challenge concerning some ferredoxins such as 3Fe/4Fe-4S or 7Fe/8Fe-8S as they differ with only one Fe-atom, and thus manual identification is needed to classify ferredoxins belonging to 2[4Fe-4S]Alv. Another challenge concerns ferredoxins assigned to one Fe-S cluster type, such as 4Fe-4S or 2[4Fe-4S], which then show inter-conversion capability to 3Fe-4S and 7Fe-8S due to an oxidative or reductive environment [45,51,52,53,54]. Assigning these ferredoxins to their correct Fe-S cluster type is still challenging. In a recent study, based on the Fe-S binding-motif pattern, canonical motifs for each of the ferredoxin Fe-S cluster types have been proposed [1]. These canonical motifs are: CX_3–5_CX_1–2_CX_22–82_C, CX_2–12_CX_30–44_CX_3_C, and CX_4–7_CX_29–35_C for 2Fe-2S; CX_5_CX_35–49_CP for 3Fe-4S; CX_2–5_CX_2–3_CX_30–45_CP for 4Fe-4S; CX_3–10_CX_3_CPX_17–40_CX_2_CX_2_CX_3_CP for 7Fe-8S; CX_2–7_CX_2–4_CX_2–3_CX_14–42_CX_1–2_CX_2–8_CX_3_C for 2[4Fe-4S]; and CX_2_CX_2_CX_3_CX_18–46_CX_2_CX_2–8_CX_3_CX_3_C for 2[4Fe-4S]Alv [1]. Such motifs assist in sorting and assigning the putative ferredoxin proteins into different cluster types. 

## 8. Conclusions and Future Perspectives

Undertaking ferredoxin classification and characterization has excellent applications in molecular biology studies, especially in identifying the evolutionarily linked ferredoxins between organisms and species and laterally/horizontally transferred ferredoxins between prokaryotes and eukaryotes. Ferredoxin subtyping also helps to understand the diversity within a ferredoxin cluster type; for example, establishing ferredoxins’ diverse roles in cellular metabolism, as observed between *Bacteroides* and *Firmicutes* [48]. Ferredoxin subtyping also shows that organisms may have ferredoxins belonging to the same cluster types, but they are diverse in function as they belong to different subtypes [1,48]. 

The current review described only a few subtype cysteine spacing signatures (CSSs) under each cluster type, as only a fraction of ferredoxins were annotated in this work concerning their subtyping. Further analysis is being carried out to identify and classify all known ferredoxins across the domains of life, especially in bacteria. Furthermore, ferredoxin databases are being developed where one can access ferredoxins’ information according to their types and subtypes. This database will also allow BLAST analysis options to be undertaken where researchers can identify the type and subtype to which a ferredoxin may belong. In the future, it would also be interesting to create super subtypes based on their percentage sequence identity, as the higher the sequence identity, the higher the chances that these ferredoxins may be involved in a similar biological process in cellular metabolic pathways with similar electron donor and receiver partners. Ferredoxin super-subtyping may help answer the challenging question: do particular ferredoxins have any unique specificity towards electron donors or receivers? This might be an interesting puzzle to solve and will assist in selecting a specific ferredoxin for the efficient transfer of electrons for biotechnologically valuable reactions. 

## Figures and Tables

**Table 1:** Cysteine spacing signature (CSS) sequences in ferredoxin subtypes. The CSS sequences (ferredoxin type, subtype, and CSS) were retrieved from published studies [1,47,48]. In the CSS sequences, C indicates a cysteine amino acid, P indicates proline, X indicates any amino acid, and the numerical number is an amino acid number.

Subtype Number	Nomenclature	Cysteine Spacing Signature (CSS)
**2Fe-2S**		
Subtype 1	2Fe-2SST1	CX_5_CX_2_CX_36_C
Subtype 2	2Fe-2SST2	CX_5_CX_2_CX_37_C
Subtype 3	2Fe-2SST3	CX_4_CX_2_CX_29_C
Subtype 4	2Fe-2SST4	CX_5_CX_2_CX_35_C
Subtype 5	2Fe-2SST5	CX_5_CX_2_CX_38_C
Subtype 6	2Fe-2SST6	CX_4_CX_2_CX_34_C
Subtype 7	2Fe-2SST7	CX_4_CX_2_CX_51_C
Subtype 8	2Fe-2SST8	CX_4_CX_2_CX_31_C
Subtype 9	2Fe-2SST9	CX_4_CX_2_CX_33_C
Subtype 10	2Fe-2SST10	CX5CX2CX_39_C
Subtype 11	2Fe-2SST11	CX_4_CX_2_CX_35_C
Subtype 12	2Fe-2SST12	CX_4_CX_2_CX_25_C
Subtype 13	2Fe-2SST13	CX_7_CX_34_CX_3_C
Subtype 14	2Fe-2SST14	CX_4_CX_29_C
Subtype 15	2Fe-2SST15	CX_7_CX_29_C
Subtype 16	2Fe-2SST16	CX_7_CX_35_C
Subtype 17	2Fe-2SST17	CX_5_CX_2_CX_33_C
Subtype 18	2Fe-2SST18	CX_5_CX_2_CX_34_C
Subtype 19	2Fe-2SST19	CX_10_CX_31_CX_3_C
Subtype 20	2Fe-2SST20	CX_5_CX_2_CX_32_C
Subtype 21	2Fe-2SST21	CX_4_CX_31_CX_3_C
Subtype 22	2Fe-2SST22	CX_2_CX_41_CX_3_C
Subtype 23	2Fe-2SST23	CX_7_CX_38_CX_3_C
Subtype 24	2Fe-2SST24	CX_4_CX_2_CX_30_C
Subtype 25	2Fe-2SST25	CX_5_CX_2_CX_30_C
Subtype 26	2Fe-2SST26	CX_12_CX_30_CX_3_C
Subtype 27	2Fe-2SST27	CX_12_CX_31_CX_3_C
Subtype 28	2Fe-2SST28	CX_8_CX_44_CX_3_C
Subtype 29	2Fe-2SST29	CX_8_CX_33_CX_3_C
Subtype 30	2Fe-2SST30	CX_8_CX_32_CX_3_C
Subtype 31	2Fe-2SST31	CX_4_CX_36_CX_3_C
Subtype 32	2Fe-2SST32	CX_8_CX_38_CX_3_C
Subtype 33	2Fe-2SST33	CX_8_CX_39_CX_3_C
Subtype 34	2Fe-2SST34	CX_9_CX_33_CX_3_C
Subtype 35	2Fe-2SST35	CX_12_CX_33_CX_3_C
Subtype 36	2Fe-2SST36	CX_3_CX_1_CX_38_C
Subtype 37	2Fe-2SST37	CX_12_CX_32_CX_3_C
Subtype 38	2Fe-2SST38	CX_4_CX_2_CX_28_C
Subtype 39	2Fe-2SST39	CX_4_CX_2_CX_46_C
Subtype 40	2Fe-2SST40	CX_4_CX_2_CX_49_C
Subtype 41	2Fe-2SST41	CX_4_CX_2_CX_45_C
Subtype 42	2Fe-2SST42	CX_4_CX_2_CX_65_C
Subtype 43	2Fe-2SST43	CX_4_CX_2_CX_50_C
Subtype 44	2Fe-2SST44	CX_4_CX_2_CX_47_C
Subtype 45	2Fe-2SST45	CX_4_CX_2_CX_48_C
Subtype 46	2Fe-2SST46	CX_5_CX_2_CX_52_C
Subtype 47	2Fe-2SST47	CX_5_CX_2_CX_31_C
Subtype 48	2Fe-2SST48	CX_5_CX_2_CX_28_C
Subtype 49	2Fe-2SST49	CX_5_CX_2_CX_27_C
Subtype 50	2Fe-2SST50	CX_5_CX_2_CX_82_C
Subtype 51	2Fe-2SST51	CX_5_CX_2_CX_29_C
Subtype 52	2Fe-2SST52	CX_4_CX_2_CX_32_C
Subtype 53	2Fe-2SST53	CX_5_CX_2_CX_42_C
Subtype 54	2Fe-2SST54	CX_4_CX_2_CX_22_C
Subtype 55	2Fe-2SST55	CX_4_CX_2_CX_27_C
Subtype 56	2Fe-2SST56	CX_4_CX_2_CX_64_C
Subtype 57	2Fe-2SST57	CX_4_CX_2_CX_39_C
Subtype 58	2Fe-2SST58	CX_4_CX_2_CX_53_C
**3Fe-4S**		
Subtype 1	3Fe-4SST1	CX_5_CX_38_CP
Subtype 2	3Fe-4SST2	CX_5_CX_37_CP
Subtype 3	3Fe-4SST3	CX_5_CX_36_CP
Subtype 4	3Fe-4SST4	CX_5_CX_40_CP
Subtype 5	3Fe-4SST5	CX_5_CX_36_CP
Subtype 6	3Fe-4SST6	CX_5_CX_35_CP
Subtype 7	3Fe-4SST7	CX_5_CX_49_CP
Subtype 8	3Fe-4SST8	CX_5_CX_32_CP
Subtype 9	3Fe-4SST9	CX_5_CX_53_CP
Subtype 10	3Fe-4SST10	CX_5_CX_54_CP
Subtype 11	3Fe-4SST11	CX_5_CX_47_CP
Subtype 12	3Fe-4SST12	CX_5_CX_46_CP
Subtype 13	3Fe-4SST13	CX_5_CX_43_CP
Subtype 14	3Fe-4SST14	CX_5_CX_56_CP
Subtype 15	3Fe-4SST15	CX_5_CX_50_CP
Subtype 16	3Fe-4SST16	CX_5_CX_52_CP
Subtype 17	3Fe-4SST17	CX_7_CX_53_CP
Subtype 18	3Fe-4SST18	CX_5_CX_48_CP
Subtype 19	3Fe-4SST19	CX_5_CX_66_CP
Subtype 20	3Fe-4SST20	CX_5_CX_58_CP
Subtype 21	3Fe-4SST21	CX_5_CX_59_CP
**4Fe-4S**		
Subtype 1	4Fe-4SST1	CX_5_CX_3_CX_33_CP
Subtype 2	4Fe-4SST2	CX_2_CX_2_CX_43_CP
Subtype 3	4Fe-4SST3	CX_2_CX_2_CX_45_CP
Subtype 4	4Fe-4SST4	CX_2_CX_2_CX_37_CP
Subtype 5	4Fe-4SST5	CX_2_CX_2_CX_44_CP
Subtype 6	4Fe-4SST6	CX_2_CX_2_CX_39_CP
Subtype 7	4Fe-4SST7	CX_2_CX_2_CX_36_CP
Subtype 8	4Fe-4SST8	CX_2_CX_2_CX_34_CP
Subtype 9	4Fe-4SST9	CX_2_CX_2_CX_38_CP
Subtype 10	4Fe-4SST10	CX_5_CX_3_CX_32_CP
Subtype 11	4Fe-4SST11	CX_5_CX_3_CX_30_CP
Subtype 12	4Fe-4SST12	CX_5_CX_3_CX_31_CP
Subtype 13	4Fe-4SST13	CX_2_CX_2_CX_48_CP
Subtype 14	4Fe-4SST14	CX_2_CX_2_CX_47_CP
Subtype 15	4Fe-4SST15	CX_3_CX_5_CX_32_CP
**7Fe-8S**		
Subtype 1	7Fe-8SST1	CX_7_CX_3_CPX_17_CX_2_CX_2_CX_3_CP *
Subtype 2	7Fe-8SST2	CX_5_CX_3_CPX_40_CX_2_CX_2_CX_3_CP
Subtype 3	7Fe-8SST10	CX_3_CX_3_CPX_22_CX_2_CX_2_CX_3_CP
Subtype 4	7Fe-8SST4	CX_10_CX_3_CPX_22_CX_2_CX_2_CX_3_CP
Subtype 5	7Fe-8SST5	CX_5_CX_3_CPX_26_CX_2_CX_2_CX_3_CP
Subtype 6	7Fe-8SST6	CX_5_CX_3_CPX_24_CX_2_CX_2_CX_3_CP
Subtype 7	7Fe-8SST7	CX_10_CX_3_CPX_17_CX_2_CX_2_CX_3_CP
Subtype 8	7Fe-8SST8	CX_5_CX_3_CPX_22_CX_2_CX_2_CX_3_CP
Subtype 9	7Fe-8SST9	CX_5_CX_3_CPX_18_CX_2_CX_2_CX_3_CP
**2[4Fe-4S]**		
Subtype 1	2[4Fe-4S]ST1	CX_2_CX_4_CX_3_CX_18_CX_2_CX_2_CX_3_C
Subtype 2	2[4Fe-4S]ST2	CX_2_CX_2_CX_3_CX_18_CX_2_CX_8_CX_3_C
Subtype 3	2[4Fe-4S]ST3	CX_2_CX_2_CX_3_CX_20_CX_2_CX_2_CX_3_C
Subtype 4	2[4Fe-4S]ST4	CX_7_CX_2_CX_3_CX_23_CX_2_CX_2_CX_3_C
Subtype 5	2[4Fe-4S]ST5	CX_2_CX_2_CX_3_CX_42_CX_2_CX_2_CX_3_C
Subtype 6	2[4Fe-4S]ST6	CX_2_CX_2_CX_3_CX_18_CX_2_CX_7_CX_3_C
Subtype 7	2[4Fe-4S]ST7	CX_2_CX_2_CX_3_CX_18_CX_2_CX_6_CX_3_C
Subtype 8	2[4Fe-4S]ST8	CX_2_CX_2_CX_3_CX_24_CX_2_CX_2_CX_3_C
Subtype 9	2[4Fe-4S]ST9	CX_2_CX_2_CX_3_CX_18_CX_2_CX_2_CX_3_C
Subtype 10	2[4Fe-4S]ST10	CX_2_CX_2_CX_3_CX_21_CX_2_CX_2_CX_3_C
Subtype 11	2[4Fe-4S]ST11	CX_2_CX_2_CX_3_CX_18_CX_3_CX_2_CX_3_C
Subtype 12	2[4Fe-4S]ST12	CX_2_CX_2_CX_3_CX_28_CX_2_CX_2_CX_3_C
Subtype 13	2[4Fe-4S]ST13	CX_2_CX_2_CX_3_CX_27_CX_2_CX_2_CX_3_C
Subtype 14	2[4Fe-4S]ST14	CX_5_CX_2_CX_3_CX_20_CX_2_CX_2_CX_3_C
Subtype 15	2[4Fe-4S]ST15	CX_2_CX_2_CX_3_CX_19_CX_2_CX_2_CX_3_C
Subtype 16	2[4Fe-4S]ST16	CX_2_CX_2_CX_3_CX_40_CX_2_CX_2_CX_3_C
Subtype 17	2[4Fe-4S]ST17	CX_2_CX_2_CX_3_CX_29_CX_2_CX_2_CX_3_C
Subtype 18	2[4Fe-4S]ST18	CX_4_CX_2_CX_3_CX_18_CX_2_CX_2_CX_3_C
Subtype 19	2[4Fe-4S]ST18	CX_3_CX_2_CX_3_CX_20_CX_2_CX_2_CX_3_C
Subtype 20	2[4Fe-4S]ST20	CX_2_CX_2_CX_3_CX_17_CX_2_CX_2_CX_3_C
Subtype 21	2[4Fe-4S]ST21	CX_3_CX_3_CX_3_CX_37_CX_1_CX_3_CX_3_C
Subtype 22	2[4Fe-4S]ST22	CX_2_CX_2_CX_3_CX_26_CX_2_CX_2_CX_3_C
Subtype 23	2[4Fe-4S]ST23	CX_2_CX_2_CX_3_CX_30_CX_2_CX_2_CX_3_C
Subtype 24	2[4Fe-4S]ST24	CX_2_CX_2_CX_3_CX_33_CX_2_CX_2_CX_3_C
Subtype 25	2[4Fe-4S]ST25	CX_2_CX_2_CX_3_CX_32_CX_2_CX_2_CX_3_C
Subtype 26	2[4Fe-4S]ST26	CX_2_CX_2_CX_3_CX_23_CX_2_CX_2_CX_3_C
Subtype 27	2[4Fe-4S]ST27	CX_2_CX_2_CX_3_CX_34_CX_2_CX_2_CX_3_C
Subtype 28	2[4Fe-4S]ST28	CX_2_CX_2_CX_3_CX_14_CX_2_CX_2_CX_3_C
Subtype 29	2[4Fe-4S]ST29	CX_2_CX_2_CX_3_CX_22_CX_2_CX_2_CX_3_C
Subtype 30	2[4Fe-4S]ST30	CX_2_CX_2_CX_2_CX_38_CX_2_CX_2_CX_3_C
Subtype 31	2[4Fe-4S]ST31	CX_4_CX_2_CX_3_CX_19_CX_2_CX_2_CX_3_C
Subtype 32	2[4Fe-4S]ST32	CX_5_CX_2_CX_3_CX_19_CX_2_CX_2_CX_3_C
Subtype 33	2[4Fe-4S]ST33	CX_2_CX_2_CX_3_CX_16_CX_2_CX_2_CX_3_C
Subtype 34	2[4Fe-4S]ST34	CX_2_CX_2_CX_3_CX_38_CX_2_CX_2_CX_3_C
Subtype 35	2[4Fe-4S]ST35	CX_3_CX_4_CX_3_CX_20_CX_2_CX_2_CX_3_C
Subtype 36	2[4Fe-4S]ST36	CX_2_CX_4_CX_3_CX_21_CX_2_CX_2_CX_3_C
**2[4Fe-4S]Alv**		
Subtype 1	2[4Fe-4S]AlvST1	CX_2_CX_2_CX_3_CX_18_CX_2_CX_8_CX_3_CX_3_C
Subtype 2	2[4Fe-4S]AlvST2	CX_2_CX_2_CX_3_CX_39_CX_2_CX_2_CX_3_CX_3_C
Subtype 3	2[4Fe-4S]AlvST3	CX_2_CX_2_CX_3_CX_43_CX_2_CX_2_CX_3_CX_3_C
Subtype 4	2[4Fe-4S]AlvST4	CX_2_CX_2_CX_3_CX_42_CX_2_CX_2_CX_3_CX_3_C
Subtype 5	2[4Fe-4S]AlvST5	CX_2_CX_2_CX_3_CX_40_CX_2_CX_2_CX_3_CX_3_C
Subtype 6	2[4Fe-4S]AlvST6	CX_2_CX_2_CX_3_CX_38_CX_2_CX_2_CX_3_CX_3_C
Subtype 7	2[4Fe-4S]AlvST7	CX_2_CX_2_CX_3_CX_46_CX_2_CX_2_CX_3_CX_3_C
Subtype 8	2[4Fe-4S]AlvST8	CX_2_CX_2_CX_3_CX_44_CX_2_CX_2_CX_3_CX_3_C
Subtype 9	2[4Fe-4S]AlvST9	CX_2_CX_2_CX_3_CX_30_CX_2_CX_2_CX_3_CX_3_C
Subtype 10	2[4Fe-4S]AlvST10	CX_2_CX_2_CX_3_CX_19_CX_2_CX_2_CX_3_CX_3_C
Subtype 11	2[4Fe-4S]AlvST11	CX_2_CX_2_CX_3_CX_52_CX_2_CX_8_CX_3_CX_3_C
Subtype 12	2[4Fe-4S]AlvST12	CX_2_CX2CX_3_CX_51_CX_2_CX_8_CX_3_CX_3_C

Note: *, only 7Fe-8S ferredoxins from *M. tuberculosis* H37Rv (Rv2007c) were found to have “arginine (R)” instead of “proline (P).” Proline is not conserved in 2[4Fe-4S] cluster ferredoxins and is thus not included in the signature sequence. Although proline was included for 7Fe-8S cluster ferredoxins, only cysteine residues and the amino acid spacing between these residues can be taken as signatures [1].
